# Predicting individual cases of major adolescent psychiatric conditions with artificial intelligence

**DOI:** 10.1038/s41398-023-02599-9

**Published:** 2023-10-10

**Authors:** Nina de Lacy, Michael J. Ramshaw, Elizabeth McCauley, Kathleen F. Kerr, Joan Kaufman, J. Nathan Kutz

**Affiliations:** 1 Huntsman Mental Health Institute, Salt Lake City, UT 84103 USA; 2 Department of Psychiatry, University of Utah, Salt Lake City, UT 84103 USA; 3 Department of Psychiatry and Behavioral Sciences, University of Washington, Seattle, WA USA; 4 Department of Biostatistics, University of Washington, Seattle, WA USA; 5 KSADS-COMP, LLC, Madison, WI USA; 6 Department of Applied Mathematics, University of Washington, Seattle, WA USA; 7 AI Institute for Dynamical Systems, Seattle, WA USA

**Keywords:** Psychiatric disorders, Neuroscience

## Abstract

Three-quarters of lifetime mental illness occurs by the age of 24, but relatively little is known about how to robustly identify youth at risk to target intervention efforts known to improve outcomes. Barriers to knowledge have included obtaining robust predictions while simultaneously analyzing large numbers of different types of candidate predictors. In a new, large, transdiagnostic youth sample and multidomain high-dimension data, we used 160 candidate predictors encompassing neural, prenatal, developmental, physiologic, sociocultural, environmental, emotional and cognitive features and leveraged three different machine learning algorithms optimized with a novel artificial intelligence meta-learning technique to predict individual cases of anxiety, depression, attention deficit, disruptive behaviors and post-traumatic stress. Our models tested well in unseen, held-out data (AUC ≥ 0.94). By utilizing a large-scale design and advanced computational approaches, we were able to compare the relative predictive ability of neural versus psychosocial features in a principled manner and found that psychosocial features consistently outperformed neural metrics in their relative ability to deliver robust predictions of individual cases. We found that deep learning with artificial neural networks and tree-based learning with XGBoost outperformed logistic regression with ElasticNet, supporting the conceptualization of mental illnesses as multifactorial disease processes with non-linear relationships among predictors that can be robustly modeled with computational psychiatry techniques. To our knowledge, this is the first study to test the relative predictive ability of these gold-standard algorithms from different classes across multiple mental health conditions in youth within the same study design in multidomain data utilizing >100 candidate predictors. Further research is suggested to explore these findings in longitudinal data and validate results in an external dataset.

## Introduction

The majority of lifetime primary mental illness is clinically diagnosed from 10 to 24 years of age (y) when incidence rises exponentially, with 50% of lifetime illness diagnosed by 14 y and 75% by 24 y [1]. Importantly, manifest illness is typically preceded by ~8–10 years of symptoms [1–4], offering a window for intervention and prevention. However, it is currently challenging to easily identify youth at elevated risk, and diagnosis tends to occur retrospectively. Since evidence across disorders suggests that early intervention improves outcomes [5, 6] and reduces resource use [7], identifying specific factors that predict individual cases would likely promote advances in population health and inform public policy. In the last decade, there has been increasing interest in using machine learning (ML) techniques to construct predictive models of youth mental illness. Here, we use the term ‘prediction’ as commonly employed in ML: to train a discriminative classifier to predict the quantitative value of a target variable by analyzing patterns in input data (‘predictors’ or ‘features’). ML techniques can offer a useful bridge between work focused on identifying statistical associations at a group level and clinical relevance since they can “generate individual predictions from multidimensional data, providing multivariate signatures that are valid at the single-subject level” [8, 9]. Further, these approaches can simultaneously analyze dozens or hundreds of candidate predictors (though this can pose the problems of feature selection and computational complexity) and incorporate non-linear relationships among a set of predictors.

Several types of datasets have historically been used to conduct a predictive classification of youth mental illnesses. Outside the US, population-level registry data may be available, offering large sample sizes (*n* > 10,000) but are often limited with respect to data types. In particular, neuroimaging and/or psychometric testing is typically not available. Examples of this type include work performed in Australia, China, Sweden and the UK [10–13]. Alternatively, some participant samples have been assembled with a broader array of data types, including neuroimaging, that are focused on individual conditions, such as the IMAGEN (depression, anxiety) or ENIGMA (ADHD) datasets [14–16]. To promote comparative discovery at scale, federal and other organizations have most recently sponsored the formation of large transdiagnostic or population-based samples that collect a wide array of multimodal data types and hundreds to thousands of descriptive variables. In peri-adolescence, flagship initiatives of this type include the ongoing population-level ABCD and Healthy Brain Network studies, the latter being used in the present study [17–19]. Such large-scale, multidomain data repositories offer the opportunity to compare the relative ability of a wide range of phenotypic, physiologic and neural candidate predictors to predict cases of multiple psychiatric illnesses in more naturalistic, community-based samples. In recent years, a new generation of studies has started to emerge applying ML predictive classification in these large, multidomain, naturalistic participant samples that offer a wide range of candidate predictor data types. To date, these have focused on comparing the predictive ability of different ML algorithms in individual conditions such as depression or disruptive behaviors and used <100 candidate predictors. Certain common mental health conditions in youth have received less emphasis in ML-based predictive work. In particular, there are very few studies aimed at predicting anxiety or post-traumatic stress (PTS) in youth using ML classifiers.

Extant studies using ML techniques to construct individual-level predictive classifiers of cases of youth mental illnesses typically compare the performance of multiple algorithm types across different classes to explore different ways of fitting models to the data, a recommended practice [20]. These have most often been linear or decision-tree algorithmic classes that offer the advantage of producing models that are inherently explainable: the identity of each of the final set of predictors is known, and their relative importance may be quantified. Typically, such studies have found that decision-tree algorithms achieved superior performance. Newer deep learning algorithms (e.g., artificial neural networks) are powerful techniques that are well-suited to high-dimensional data. However, they have been less often employed to construct discriminative classifiers in psychiatry and are ‘black box’ methods that use intermediate features that are not interpretable by humans. While all ML algorithms have hyperparameter settings that govern learning that require ‘tuning’ (a principled method to optimize hyperparameter settings for performance), deep learning algorithms are also notoriously challenging to tune. Few studies to date have compared the relative effectiveness of deep learning versus linear and decision-tree-based algorithms in constructing predictive (classification) models of youth mental illness cases. Most, but not all, of those that have done so have found that deep learning has outperformed [12, 13, 16, 21]. Moreover, no studies to our knowledge compare the relative predictive ability of different ML algorithmic classes across multiple mental health conditions in youth within the same participant cohort and study design.

Anxiety, attention deficit, depression, disruptive behaviors and post-traumatic stress (PTS) are among the most common and disabling mental illnesses in youth. The emphasis of the present study was to enlarge our knowledge of the ability of ML algorithms to provide interpretable predictive models of these major mental illnesses in youth by (1) analyzing a larger number of candidate predictors (*n* = 160) of multiple data types (e.g., psychosocial, cognitive and neural); (2) comparing the predictive ability of gold-standard ML algorithms from different classes (deep, decision tree and logistic regression classifiers); (3) construct interpretable models across multiple mental health conditions within the same analytic design; and (4) using a large, naturalistic participant sample that resembles that presenting at mental health clinics, i.e., is enriched for at least one behavioral complaint.

We hypothesized that deep learning optimized with artificial intelligence (AI) would outperform decision-tree and logistic (linear) regression in constructing individual-level psychiatric cases of major mental illnesses during the peri-adolescent developmental life stage. To determine the relative performance of different algorithm classes in high-dimension multidomain data, we compared the predictive ability of gold-standard logistic regression (LR), decision tree (XGBoost: XGB) and deep learning (artificial neural network: ANN) algorithms to predict individual cases of anxiety, attention deficit, depression and disruptive behaviors in a large (*n* = 1120), naturalistic, transdiagnostic sample of youth aged 5–21 y and their parents from the Healthy Brain Network (HBN) cohort. In addition, we added an exploratory analysis that attempted to predict PTS, which has not historically been a focus of ML techniques. Predictive models were constructed that simultaneously surveyed and quantified the relative predictive ability of 160 candidate predictors encompassing neural, prenatal, developmental, physiologic, sociocultural, environmental, emotional and cognitive features. We did not stratify the sample in order to test the predictive ability of this design and algorithms in more naturalistic data. By nesting each ML algorithm (LR; XGB; ANN) inside an innovative AI meta-learning method, we optimized algorithmic performance by jointly learning hyperparameters and performing automated, principled feature selection while also rendering deep learning interpretable for translational applications. All models were tested in a held-out test dataset, and all results presented in this manuscript are from testing in the held-out test set. To our knowledge, this is the first study to test the relative predictive ability of these gold-standard algorithms from different classes across multiple mental health conditions in youth within the same study design in multidomain data and to incorporate PTS related to accumulated early life adverse events in a ML-based predictive classification.

## Materials and methods

### Terminology and definitions

This manuscript uses ML terms and conventions throughout [20, 22, 23]. Accordingly, ‘prediction’ refers to predicting the quantitative value of a target variable by analyzing patterns in input data (‘candidate predictors’ or ‘features’) used to predict the value of the target variable. In this case, input data is chronologically contemporaneous with target variables since this is a cross-sectional participant sample. We neither aim to predict cases using antecedent features nor predict the future occurrence of cases in individuals who are not manifesting symptoms. We refer to the set of all input data as containing ‘candidate predictors’ (features) and the actual predictors identified in final, optimized models as ‘final predictors’. The set of observations (*n* = 784) used to train, fit and test models under development is referred to as the ‘training set’, and the unseen held-out set of observations (*n* = 336) is termed the ‘test set’. In terms of performance, we use ‘generalizability’ to refer to the ability of a trained model to adapt properly to new, previously unseen data drawn from the same distribution as the one used to create the model, though we acknowledge that generalization can have a different meaning in other fields. ‘Precision’ refers to the fraction of positive predictions that were correct; ‘Recall’ to the proportion of true positives that are correctly predicted; and ‘Accuracy’ to the number of correct predictions as a fraction of total predictions. We provide Receiver Operating Characteristic curves (ROC Curves) showing the performance of classification models at different classification thresholds plotting true positive versus false positive rates, and the Area Under the Curve (AUC), defined as the two-dimensional area under the ROC curve from (0,0) to (1,1).

### Participant sample

**Fig. 1:**
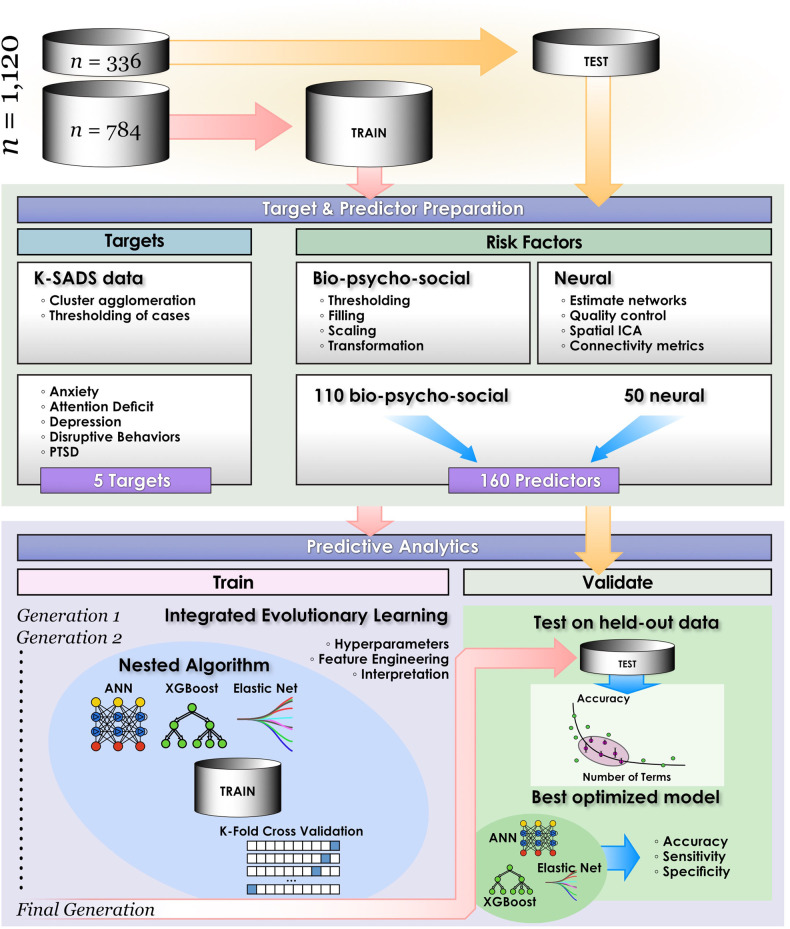
Computational pipeline. The study computational pipeline is shown for classification experiments. Individual machine learning algorithms (artificial neural networks, XGBoost and logistic regression) are nested within an integrated evolutionary learning architecture to optimize learning by providing joint feature selection and optimization across the hyperparameters. This figure is adapted from de Lacy et al., Integrated Evolutionary Learning: an artificial intelligence approach to joint learning of features and hyperparameters for optimized, explainable machine learning. *Front Artificial Intelligence* (2022).

Participants with at least one complete resting-state fMRI scan (365 volumes) and available phenotypic data were selected from the ongoing Healthy Brain Network (HBN) study to Release 8 [17]. The HBN initiative collects multidomain data from youth with at least one behavioral concern aged 5–21 y in the New York City area. This is a naturalistic, cross-sectional community-based population sample and not a fully representative epidemiologic design or longitudinal sample. No attempt was made in the original or present study to stratify the sample with respect to demographic or psychiatric features or determine the point of disease onset for any mental health condition. Exclusion criteria are the presence of acute safety concerns, cognitive or behavioral impairment (e.g., IQ level <66) or medical concerns that might confound brain imaging. The overall and present study sample is enriched for mental health concerns: approximately 2/3 meet the criteria for a clinical diagnosis. Demographic features of this participant sample are presented in Table 1, as well as summary metrics regarding participants’ level of autism traits, handedness, body mass index and performance in core metrics of cognitive and executive function. The participant sample was randomly split, with 70% used as the training/validation set (784 participants) and 30% (336 participants) reserved as an unseen test set. The number and proportion of subjects with cases of each mental health disorder (predictive target) in the study may be viewed in Table 2. The data preparation and preprocessing pipeline were applied separately to the training and test sets (Fig. 1). The HBN study was approved by the Chesapeake Institutional Review Board. The present study was deemed not human subjects research by the University of Washington Institutional Review Board and the University of Utah Institutional Review Board.

**Table 1:** Characteristics of participant sample.

Characteristic	Range	Mean	Median
Age in years	5.1–21.5	10.8	9.9
Full Scale Intelligence Quotient	42–147	98.2	100
Body mass index	12.3–40.3	19.3	18.2
Autism traits	0–47	7.2	4
Handedness	(−100)– (+100)	59.4	77.8
Dimensional change	0–100	34.2	25.0
Inhibitory control	0–99	26.2	19.0
Working memory	0–100	41.3	37.0
Pattern recognition	0–100	39.0	32.0

Characteristics of 1120 participants in the study are shown. The sample contained 729 male youth and 391 female youth. Full scale intelligence quotient was determined with the Wechsler Intelligence Scale (WISC 5). Level of autism traits was determined with the Autism Spectrum Screening Questionnaire (ASSQ). Handedness was determined with the Edinburgh Handedness Inventory, where a score of −100 represents maximal left dominance and +100 maximal right dominance. For cognitive measures, dimensional change was assessed with a card sort task, inhibitory control with a flanker task and working memory with a list sorting task.

**Table 2:** Case counts of major mental illnesses in youth in participant sample.

	Anxiety	Attention deficit	Depression	Disruptive behaviors	PTSD
Training: cases	338, 43.1%	546, 69.7%	95, 12.1%	238, 30.4%	126, 16.1%
Training: not cases	446, 56.9%	238, 30.3%	689, 87.9%	546, 69.6%	658, 83.9%
Training: cases *after SMOTEENN*	197, 80.1%	50, 9.9%	683, 78.0%	456, 81.0%	643, 85.6%
Training: not cases *after SMOTEENN*	49, 19.9%	457, 90.1%	193, 22.0%	107, 19.0%	108, 14.4%
Test: cases	145, 43.1%	251, 74.7%	51, 15.2%	101, 30.0%	17, 5.1%
Test: not cases	191, 56.9%	85, 25.3%	285, 84.8%	235, 70.0%	319, 94.9%

Counts and percentages of cases versus not-cases are shown for anxiety, attention deficit, depression, disruptive behaviors and post-traumatic stress in the training and test data partitions in the participant sample. For the training partition, data is shown before and after synthetic oversampling with the SMOTEENN algorithm. Oversampling was not conducted in the test partition.

### Data and data preparation

#### Data collection

All data was originally collected by the Child Mind Institute as part of the ongoing HBN study. Comprehensive details of the HBN project may be accessed at the project data portal: https://fcon_1000.projects.nitrc.org/indi/cmi_healthy_brain_network/index.html. In brief, multidomain data are collected in four study visits of 3 h, each comprising cognitive and language testing, behavioral assessment and measures of family structure, stress and trauma, physical function and substance use. Cognitive testing, biological sampling and mock scanning are performed on the first visit, MRI scans on the second visit, learning and language testing on the third visit and mental health assessment on the final visit. All data are collected by the Child Mind Study Team. Further details of the data collection protocol may be accessed at: https://fcon_1000.projects.nitrc.org/indi/cmi_healthy_brain_network/The%20Project%20Plan.html.

#### Phenotypic candidate predictor preparation

**Fig. 2:**
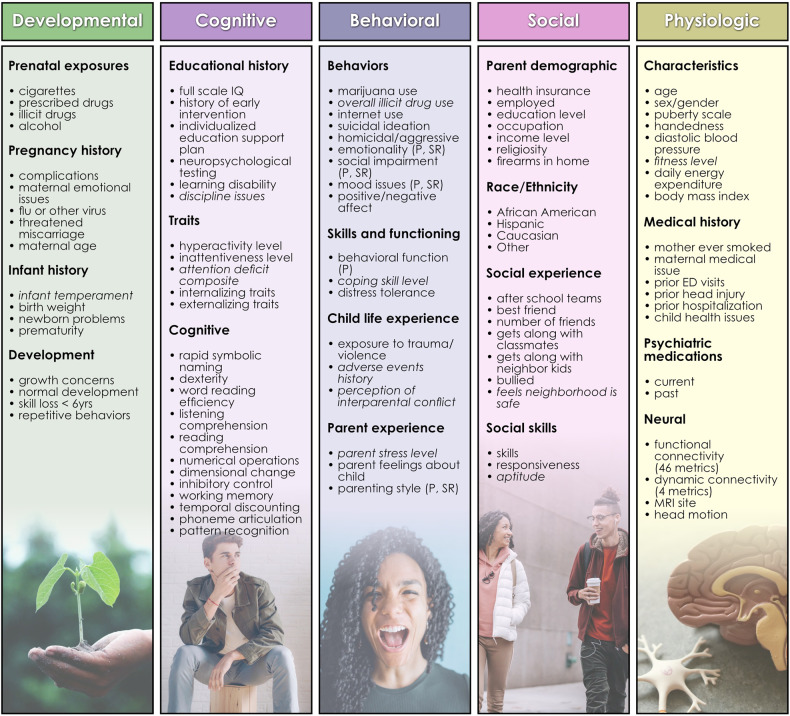
Candidate predictors for youth mental illness before and after adaptive selection with AI. 160 candidate predictors across multiple domains of human function were simultaneously assessed for their ability to predict mental illnesses. This figure is adapted from de Lacy et al., Integrated Evolutionary Learning: an artificial intelligence approach to joint learning of features and hyperparameters for optimized, explainable machine learning. *Front Artificial Intelligence* (2022).

Candidate predictors used in the present study may be reviewed in Fig. 2. Phenotypic candidate predictors include multiple metrics incorporating information about participants’ developmental history, educational history, cognitive traits and function, behavioral function, youth and parent experience, youth and parent demographics, youth social skills and function, physiologic characteristics and medical history including psychiatric medications used (current and past). As detailed above, this is a naturalistic, community-based sample, and as such, stratification on the basis of demographic features (e.g., race/ethnicity) is not performed. Typically, ML predictive classification in similar study designs takes demographic characteristics such as race and sex/gender into account by including them as candidate predictors. Similarly, a rich set of parent and youth demographic features, including race and sex, is incorporated as candidate predictors equally across all analyses. Supplementary Table 1 provides more detailed information about each candidate predictor, including the name and description of each feature and the name of the original psychometric or psychological assessment instrument from which the feature was drawn. Supplementary Table 2 (provided by the Child Mind Institute) includes a more detailed description of each original psychometric or psychological assessment instrument and the foundational literature citation pertaining to each instrument, including metrics of its psychometric properties where applicable. Candidate phenotypic predictors (*n* = 26) with >40% missing values were discarded. This threshold was used since prior research shows that good results may be obtained with ML methods with imputation up to a threshold of 50% missing data [24]. Continuous variables were trimmed to mean ± 3 standard deviations to remove outliers. Missing values were imputed using non-negative matrix factorization (NNMF). NNMF is a mathematically proven imputation method that minimizes the cost function of missing data rather than assuming zero values. It is effective at capturing both global and local structure in the data, and it has been demonstrated to perform well regardless of the underlying pattern of missingness [25–27]. Supplementary Table 3 shows the number and percentage of observations that were trimmed and filled with NNMF for the training and test sets, respectively. For continuous measures, we selected summary or total metrics. For 11 instruments (Italicized in Fig. 2), we computed the summary measure by applying feature agglomeration to recursively merge individual items and generate a single continuous measure. All features were then scaled using the MinMaxScaler. Features with non-normal distributions were transformed with the Quantile and Power transforms, and the post-transform feature version most resembling a normal distribution was selected. The univariate odds ratio and confidence interval of each candidate phenotypic predictor with each target were computed and may be viewed in Supplementary Table 4.

#### Neural connectivity features

We computed gold-standard brain functional connectivity measures from functional MRI (fMRI). Multiband 3T resting-state, eyes open fMRI comprising 365 volumes are acquired at 2 sites. After removing the first 10 volumes, each participant’s scan was realigned, coregistered, normalized and smoothed at 6 mm full width at half maximum using standard algorithms in SPM12 (https://www.fil.ion.ucl.ac.uk/spm/software/spm12/). Scans were then submitted to quality control by computing correlation with a group mask, and 21 participants with <90% correlation with this group mask were eliminated. Head motion was computed for each participant with the DVARS metric [28], and scan site and DVARS were included as candidate predictors. We used an established pipeline to perform group spatial ICA and extract a whole-brain parcellation scheme representing 15 functional components [29] with the Group ICA of fMRI Toolbox (GIFT: https://trendscenter.org/software/gift/). Components estimated by ICA were sorted into gray-matter intrinsic functional networks versus artifactual noise components with a combination of expert visual inspection by NdL and the quantitative metrics of fractional amplitude of low-frequency fluctuations and dynamic range [29]. Components with poor overlap with cerebral gray matter or low spectral metrics were discarded, and we retained a set of 10 functional intrinsic neural networks (IN). We constructed a spatial map for each IN following an established GIFT pipeline [29]. To determine functional connectivity features among INs, we computed Pearson correlations among each possible pair of spatial maps. Pearson correlations among functional networks are subsequently Fisher-transformed [*z* = atanh(*k*)] to smooth outliers. An additional set of dynamic connectivity metrics was computed by delineating stable dynamic whole-brain connectivity states from the fMRI ICA timecourses and applying the k-means clustering algorithm to connectivity windows using an established sliding window method [30]. A sliding window length was selected based on prior work demonstrating that a window length of 40–60 s produces reasonable and robust results [30–32]. These dynamic connectivity metrics describe the fluidity and range with which participants traverse brain states and are available for each individual participant. Specifically, they are the number of brain states traversed, the number of times a subject switches between brain states, the maximal L1 span achieved between brain states and total distance traveled (sum of all L1 distances). Nuisance regressors of scanner site, DVARS statistic and six realignment parameters and their six first derivatives for each participant were regressed from all connectivity models using the general linear model prior to computing. The univariate odds ratio and confidence interval of each candidate neural predictor with each target were computed and may be viewed in Supplementary Table 4. More details of specific neural metrics may be found in Supplementary Table 1.

### Predictive targets

Predictive targets of mental illness cases were computed with data obtained from the computerized version of the Kiddie Schedule for Affective Disorders and Schizophrenia (KSADS-COMP) [33, 34], a standardized, semistructured diagnostic interview administered by a clinician to youth participants and their parent(s) used widely in youth psychiatric research. Responses for the diagnostic screening assessment were used covering multiple symptoms across all major mental illnesses obtained through clinician consensus. For each of the five illness targets in this study, the multiple KSADS symptom scores available for each diagnostic class were reduced to a single metric using feature agglomeration. The distribution of scores for each illness target was then determined, and participants were thereby divided into ‘cases’ and ‘not cases’ to form a binary target vector for classification. Specifically, the group of participants who exhibited a zero agglomerated score on KSADS symptoms were deemed ‘not cases’. The second group exhibited scores reflecting a range of symptom severity for each target, and members of this group were deemed ‘cases’. Given the naturalistic, community-based nature of this sample, which was not stratified, there was an imbalance in terms of cases versus not-cases. Synthetic oversampling with the SMOTEENN algorithm was performed on the training/validation portion of the participant sample for each target but not the test partition. The number and proportion of participants in each category (cases versus not-cases) before and after synthetic oversampling in the training set may be viewed in Table 2.

### Overview of predictive analytics

We compared the ability of three leading ML techniques to predict cases of each type of mental illness using 160 candidate predictors (Fig. 2): deep and decision tree-based learning and logistic regression. All ML estimators used (Adam; XGBoost; Logistic Regression) are ‘off the shelf’ algorithms from standard libraries (Tensorflow, Sci-Kit Learn). To optimize learning performance, each ML model was performed with k-fold cross-validation and nested within the Integrated Evolutionary Learning (IEL) AI algorithm. IEL is an AI meta-learning genetic algorithm that wraps around each ML algorithm to optimize the latter’s performance. Each ML algorithm and IEL are therefore separate but interacting computational processes: the ML algorithm fits a model and performs the classification of cases while IEL jointly learns features (predictors) and hyperparameters for each ML algorithm. Typically, ~40,000 ML model fits are pursued by IEL during each optimization process. A number of methodologic and design features were incorporated to reduce bias, and these are detailed above and below. In brief, missing data was filled using non-negative matrix factorization; preprocessing is performed separately on training and testing partitions; large-scale feature selection is performed through a principled AI meta-learning architecture; thousands of model fits are explored to enlarge the solution space; and training is terminated based on a principled basis quantified with an information theoretic fitness function. Code for the predictive analytics may be accessed at the de Lacy Laboratory GitHub: https://github.com/delacylab/integrated_evolutionary_learning.

#### Deep learning with artificial neural networks

We trained artificial neural networks using the Adam algorithm with 3 layers, 300 neurons per layer, early stopping (patience = 3, metric = validation loss) and the Relu activation function. The last output layer contained a conventional softmax function. The Adam algorithm was selected based on its established computational efficiency and suitability for problems with a large number of parameters like our study [35]. Learning parameters (Supplementary Table 5) were tuned with IEL Deep learning models were encoded with TensorFlow embedded in custom Python code.

#### Gradient-boosted tree-based learning

We trained decision tree-based models to predict mental illness cases with the XGBoost algorithm using the gbtree booster [36, 37]. This is an ensemble-based method that generates a multitude of decision trees that ‘vote’ on a composite prediction. It is accurate, resistant to overfitting when properly tuned and uses model residuals (actual–predicted values) to penalize leaves that do not improve predictions, reducing bias as well as variance. Empirically, gradient-boosted techniques have been highly successful. Hyperparameters were tuned with IEL and may be viewed in Supplementary Table 5. XGBoost was encoded with the Scikit-Learn wrapper in custom Python code.

#### Logistic regression

We trained linear models to classify mental illness cases with logistic regression regularized with the ElasticNet [38]. The latter regularization method linearly combines the L1 penalty of the LASSO (least absolute shrinkage and selection operator) and the L2 penalty of the Ridge method. It produces superior results in real-world and simulated data, particularly to the use of LASSO alone. For the logistic regression analysis, the odds ratio and confidence interval of the optimized model were computed. The L1 and L2 parameters were tuned using IEL (Supplementary Table 5). We encoded logistic regression models using the Scikit-learn algorithm embedded within custom Python code.

#### Integrated evolutionary learning for machine learning optimization

ML models, particularly deep learning models, are famously difficult to ‘tune’, i.e., determine the right values of the many algorithm hyperparameters (settings) that control learning and can have a dramatic effect on performance. To achieve the best performance of ML experiments, we developed and here applied a meta-learning technique we call Integrated Evolutionary Learning. We have previously demonstrated that IEL improves the performance of ML predictive algorithms in comparable data by up to 20–25% versus the use of default model hyperparameters and conventional designs [39].

Typically, tuning is often done manually via ‘rules of thumb’ and ≤50 model fits are explored, introducing the possibility of bias and limiting the solution space [40–42]. Furthermore, powerful techniques such as deep learning with artificial neural networks can behave as ‘black boxes’ that obtain predictions with machine-generated intermediate features that are not interpretable by humans. For the translational or mechanistic applications that are common in biomedicine, explainable ML is a priority. Many researchers forego deep learning to focus on inherently explainable techniques like decision trees or linear models. In contrast, IEL provides adaptive, automated feature selection and principled hyperparameter tuning for any ML technique by leveraging evolutionary algorithms, advanced computational metaheuristics, instantiating the concepts of biological evolutionary selection in computer code. All results, including those obtained from deep learning, are fully explainable. IEL ‘breeds’ optimized models adaptively over hundreds of generations (Fig. 1) by selecting for improvements in a fitness function (here, the Bayes Information Criterion).

For each learning technique, we initialized the first generation of 100 models with randomized hyperparameter values or ‘chromosomes’. These were subsequently recombined, mutated or eliminated over successive generations. In recombination, ‘parent’ hyperparameters were averaged to form ‘children’. In mutation, hyperparameter settings were shifted. The range of possible values is shown in Supplementary Table 5. Excepting the logistic regression model (which has naturally bounded hyperparameter intervals in [0–1]), these were generously set to allow for broad exploration of the potential solution set. After training the initial 100 models, we computed the BIC for each solution. Of the 80 best models, 40 were recombined by averaging the hyperparameter setting after a pivot point at the midpoint to produce 20 ‘child’ models. 20 were mutated to produce the same number of child models by shifting the requisite hyperparameter by the mutation shift value (Supplementary Table 5). The remaining 20 were discarded. The next generation of models was formed by adding 60 new models with randomized settings and adding these to the 40 child models retained from the initial generation. Thereafter, an automated process continued to recombine, mutate and discard 100 models per generation in a similar fashion to minimize the BIC until the latter fitness function plateaued.

A further major issue in analyzing multidomain ‘big data’ with AI is feature selection: how to identify a small number of predictive risk factors from the much larger set under consideration in a principled manner. Empirically, models with fewer variables are simpler to train, run and understand and generalize better. As with tuning, many practitioners still use manual or semi-manual approaches. Given the large number of features screened during training, we incorporate automated feature selection within IEL to mitigate the risk of overfitting. Here, a random number of features in the range (2–50) was set for each model in the initial generation and randomly sampled from the set of 160 possible candidate predictors. After computing the BIC, feature sets from the best-performing 60 models were individually allocated to the recombined and mutated child models. Other feature sets were discarded. As with hyperparameter tuning, this process was repeated for succeeding generations until the BIC plateaued.

To facilitate computationally efficient modeling, IEL implements recursive learning. After training models until the BIC plateaued, we determine the elbow of the fitness function plotted versus the number of features. The feature set available after the warm start is constrained to the subset of features, thresholded by their importance, corresponding to the fitness function elbow. After the warm start, learning proceeds by thresholding features available for learning at increasing thresholds of the original warm start feature importance + 2-10 standard deviations. In addition, we reduce the number of models per generation to 50, with 20 models recombined and 10 models mutated. Otherwise, after restarting the training process at the warm start threshold ranges an initial generation of models was randomly initialized and training completed using the same principles as detailed above.

#### Cross-validation

For each of the three learning techniques, individual models were fit using stratified *k*-fold cross-validation, i.e., every one of the 100 models in each learning generation within IEL was individually trained and validated using cross-validation. Since the number of features for each model fit could differ within each model in every generation of IEL, *k* (the number of splits) was set as the nearest integer above [sample size/number of features]. Cross-validation was implemented with the scikit-learn StratifiedKFold function.

#### Determining predictor importance

Two types of techniques were used to compute the relative importance of each predictor in making predictions. First, Shapley Additive Explanations (SHAP) values were computed using the SHAP toolbox (https://shap.readthedocs.io/en/latest/). SHAP is a game theoretic approach that may be used to explain the output of any ML model, including ‘black box’ estimators such as artificial neural networks [43]. It unifies prior methods such as LIME, Shapley sampling values and Tree Interpreter. Second, the inherent method available for each algorithm was employed. For XGBoost, the importance of each feature was computed within the algorithm, which for decision-tree estimators is essentially a permutation-based method. To offer comparability, we also therefore determined feature importance for the artificial neural networks by embedding eli5 (https://eli5.readthedocs.io/en/latest/index.html), an established permutation algorithm, within IEL [44]. Where relevant, the relative importance of each risk factor for logistic regression was determined by computing its linear coefficient (beta).

#### Testing in held-out test data

**Fig. 3:**
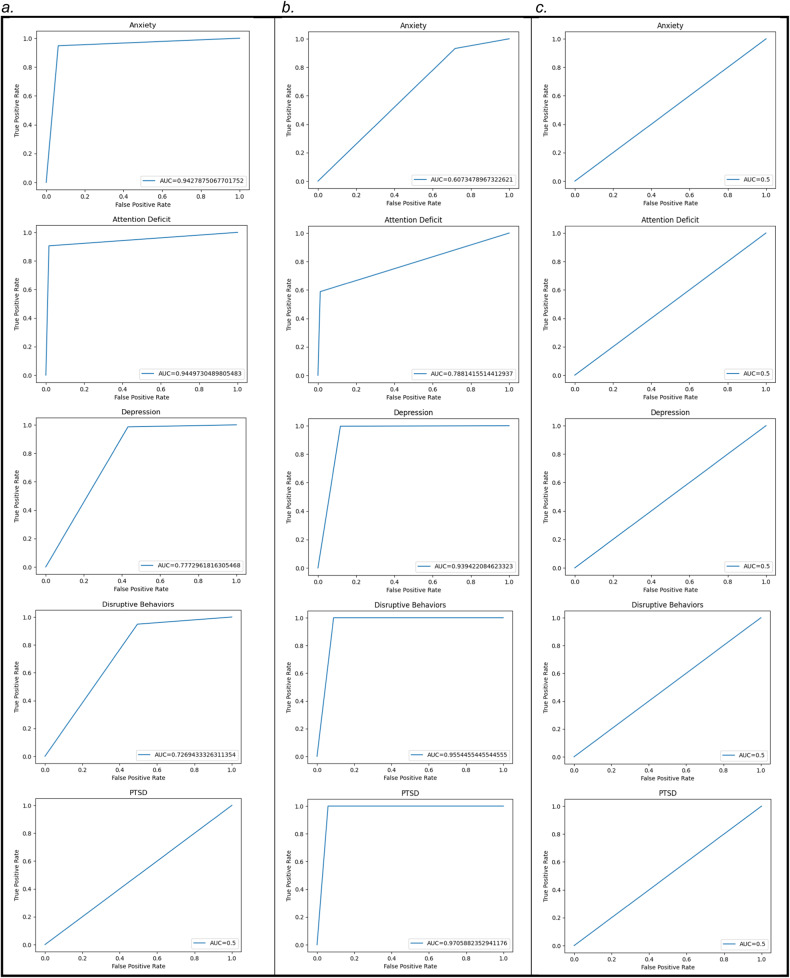
ROC curves for predicting youth mental illness cases with three machine learning algorithms. ROC curves are shown for the predictive models for **a** Deep learning with the Adam estimator; **b** Decision-tree learning with the XGBoost algorithm; and **c** Logistic regression with ElasticNet regularization, all used to predict cases of anxiety, attention deficit, depression, disruptive behaviors and post-traumatic stress in youth.

After training was completed for each type of psychiatric condition, the best-performing 100 tuned models from the IEL process were tested on the reserved unseen test set of 336 participants and accuracy, precision, recall and the AUC were determined using standard Sci-Kit learn libraries. The threshold for prediction probability was 0.5, and ROC curves are provided for each algorithm type and each target mental health condition in Fig. 3. For testing, we applied optimized model parameters (hyperparameter settings and selected features) obtained from the best-performing models of the IEL training/validation process to unseen data. Synthetic oversampling was not used to formulate predictive targets (see: “Predictive Targets”). All reported results in this paper are from testing in unseen data.

## Results

### Deep and tree-based learning optimized with IEL can predict major mental illnesses with high fidelity

We nested three gold-standard algorithms with cross-validation within the IEL architecture to compare their predictive ability: deep learning with artificial neural networks, decision tree-based learning with XGBoost and a logistic regression model regularized with the ElasticNet. The comparative ability of each technique to predict mental illness cases in youth in replication testing is shown in Table 3 in terms of accuracy, precision, recall and the AUC. ROC curves may be inspected in Fig. 3. Odds ratios and confidence intervals for the logistic regression analysis may be viewed in Supplementary Table 6.

**Table 3:** Relative performance of three algorithm types in predicting major mental illnesses in youth.

a.				
Disorder	Accuracy (%)	Precision (%)	Recall (%)	AUC
Anxiety	94.6	93.5	95.3	0.94
Attention deficit	96.4	88.5	90.1	0.95
Depression	92.6	93.4	97.5	0.77
Disruptive behaviors	84.5	84.5	94.0	0.73
PTS	95.6	95.5	100.0	0.50

b.				
Disorder	Accuracy	Precision	Recall	AUC
Anxiety	65.2	38.7	28.3	0.61
Attention deficit	89.0	72.3	96.0	0.79
Depression	97.9	13.6	88.2	0.94
Disruptive behaviors	97.3	27.7	91.1	0.96
PTS	99.7	0.05	94.1	0.97

c.				
Disorder	Accuracy	Precision	Recall	AUC
Anxiety	56.9	43.2	0.0	0.50
Attention deficit	74.7	74.7	100.0	0.50
Depression	84.8	15.2	0.0	0.50
Disruptive behaviors	70.0	30.0	0.0	0.50
PTS	95.0	0.06	0.0	0.50

The relative classification performance of **a** Deep learning with artificial neural networks; **b** Gradient-boosted tree-based learning and **c** Linear model with ElasticNet tuned with Integrated Evolutionary Learning. Performance metrics for the best-performing model for each algorithm type in testing in the held-out, unseen test set are shown.

There is no single metric that determines the best performance in an ML classifier since how performance is judged may be dependent on the use case for which the model is constructed. For example, precision (positive predictive value) may be prioritized during genetic testing, whereas recall (sensitivity) might be most important in a mass screening assay. However, the AUC is often used as an aggregate measure of overall performance across all possible classification thresholds. In terms of the AUC, we found that deep learning with artificial neural networks provided superior performance in predicting anxiety and attention deficit, whereas decision-tree learning did better at predicting depression, disruptive behaviors and PTS. Logistic regression consistently underperformed and achieved an AUC of 0.5 across all conditions, indicating these classifiers performed no better than random chance. In terms of other performance statistics, deep learning performed consistently strongly across all mental health conditions achieving ≥85% accuracy, precision and recall throughout, though its performance must be approached with caution in the case of PTS, given the AUC = 0.5. Decision-tree learning was a generally strong performer in terms of accuracy and recall in predicting attention deficit, depression, disruptive behaviors and PTS though not anxiety, but displayed low recall throughout, with the exception of attention deficit. The logistic regression technique achieved reasonable accuracy ranging from 57 to 95% over the five conditions but had notable weaker precision and recall with the exception of attention deficit, and all these results must be interpreted with caution given the AUC of 0.5 for all conditions. Interestingly, our results taken together demonstrate that attention deficit appeared the most tractable target for all approaches, including LR.

### Each major mental illness was predicted by a unique combination of final predictors

In the best-performing models produced by deep and decision-tree learning as measured by AUC, each youth mental illness had a unique combination of final predictors (Table 4). Logistic regression models contained only a single final predictor. With the exception of the exploratory PTS models, deep learning models were generally the most complex, i.e., contained the largest number of final predictors. With the exception of speech phoneme articulation in depression, physiologic, cognitive and neural metrics derived from functional MRI were de-emphasized as final predictors in optimized models, where the majority of predictors were psychosocial or psychometric indicators. In terms of the relative importance of different predictors, importance scores from the Shapley and permutation methods were in broad concordance across deep and decision-tree models.

**Table 4:** Relative importance of final predictors in predicting individual cases of major mental illnesses in youth.

	Anxiety		Attention deficit		Depression		Disruptive behaviors		PTS	
	Predictor	Imp.	Predictor	Imp.	Predictor	Imp.	Predictor	Imp.	Predictor	Imp.
ANN	Adverse life events Internalizing traits Externalizing traits Parent/child stress level Emotionality Social skills	0.25/0.09 0.24/0.14 0.23/0.11 0.21/0.080.21/0.090.20/0.07	Hyperactivity Parent feelings about child Handedness Parent/child stress level Autism traits Externalizing traits	0.21/0.17 0.21/0.08 0.14/0.04 0.12/0.05 0.11/0.04 0.08/0.03	Age Mood Speech articulation Parent feelings about child Externalizing traits	0.09/0.06 0.07/0.06 0.07/0.03 0.06/0.03 0.05/0.04	Externalizing traits Hyperactivity Social responsive-ness Mood	0.28/0.29 0.14/0.14 0.12/0.06 0.10/0.05	Parent education level Adverse life events Parent feelings about child	0.02/0.01 0.01/0.04 0.01/0.01
XGB	Adverse events Internalizing trait	0.74/0.08 0.26/0.21	Externalizing traits Hyperactivity Gets along with classmates MRI Site	0.41/0.15 0.23/0.09 0.19/0.05 0.17/0.04	Mood Negative affect	0.56/0.13 0.44/0.11	Behavioral impairment level Externalizing traits Hyperactivity	0.40/0.17 0.35/0.16 0.25/0.12	Daily life function level Normal development Coping skills level Mood	0.33/0.03 0.28/0.01 0.21/0.02 0.18/0.02
LR	Internalizing traits		Hyperactivity		Age		Externalizing traits		Mood	

Final predictors for individual cases of anxiety, attention deficit, depression, disruptive behaviors and post-traumatic stress in youth obtained via deep learning with the Adam estimator (ANN); decision-tree learning with the XGBoost algorithm (XGB); and logistic regression with ElasticNet regularization (LR) after optimization with Integrated Evolutionary Learning. The relative importance of each final predictor to making the prediction is displayed as computed via permutation with the eli5 algorithm (first number) and the Shapley Additive Explanations method using the SHAP algorithm (second number).

Anxiety was predicted in the stronger-performing deep learning model by six predictors: adverse events, externalizing traits, parent stress level, internalizing traits, emotionality and social skills aptitude. These were fairly equal in importance measured by either permutation or Shapley values. The weaker-performing decision-tree algorithm used only two of these (adverse advents and internalizing traits) and the logistic regression model only internalizing traits as final predictors. In attention deficit, the deep learning model was again complex with six final predictors (hyperactivity and externalizing traits, parent-child relationship stress levels and parent feelings toward their child, handedness and autism traits). Both permutation and Shapley values more strongly weighted hyperactivity and parent feelings about their child. The decision-tree model with lower recall and AUC also selected hyperactivity and externalizing traits but added the prosocial factor of ‘gets along well with classmates’ and MRI site. The logistic regression model contained only hyperactivity. In the depression models, mood and negative affect were selected as equally weighted final predictors by the decision-tree algorithm for a higher AUC, where the deep learning model added age, speech articulation, parent feelings about their child and externalizing trait level to mood to achieve higher precision. The logistic regression model used only age. Predictors of disruptive behaviors by the decision-tree model were led by the youth’s level of behavioral impairment as well as externalizing trait and hyperactivity levels. The deep learning model also heavily weighted externalizing and hyperactivity trait levels but added social responsiveness and mood symptom levels. The logistic regression model used only externalizing traits. As reviewed above, our exploratory PTS models cannot be considered reliable in the case of deep learning or logistic regression, given their AUC = 0.05. However, the decision-tree model performed well (with the exception of low precision). Here, the model identified daily life function level, degree of normal development and levels of coping skills and mood symptoms as final predictors.

### Optimized models were parsimonious

IEL incorporates robust automated feature selection, adaptively selecting the best-performing features based on a fitness function. Our results demonstrate that only a small number of final predictors were required by either deep or decision-tree learning to generate high-fidelity predictions that generalized to unseen, held-out data across all mental illnesses, reduced from the 160 originally included for consideration. In each case, ~40,000 model fits were explored during training in the course of fitting the final, optimized model that was selected for testing in held-out, unseen data. No final model (Table 4) required more than six final predictors to make high-quality predictions (deep learning for anxiety and attention deficit and decision-tree learning for depression, disruptive behaviors and PTS) as measured by accuracy, precision, recall and the AUC.

## Discussion

Using the same analytic design across five common conditions in youth, we show that rigorous individual case predictions (AUC ≥ 0.94) can be obtained from gold-standard ML classification algorithms in a naturalistic participant sample by applying AI to optimize performance.

Anxiety and depression are common disorders in peri-adolescent youth that may overlap phenomenologically and epidemiologically and are sometimes grouped as ‘internalizing’ disorders. We found that anxiety and depression had different sets of final predictors, helping disambiguate these conditions. XGBoost best predicted depression with mood and negative affect (AUC = 0.94; Accuracy = 98%; Precision = 14%; Recall = 88%), though our results with deep learning (AUC = 0.77; Accuracy = 93%; Precision = 93%; Recall = 98%) are of value given the higher precision and recall of the latter algorithm. Here, depression was predicted by age, mood, speech articulation, how parents felt toward their child and externalizing traits. Commonalities between our findings and final predictors in prior studies (both cross-sectional and longitudinal) are in highlighting the predictive role of mood symptom level and factors related to parents or the parent-child relationship. In a large (*n* > 6000) cross-sectional sample of Australian youth, Haque et al. used 62 final features (primarily psychosocial and symptom-based) and several types of decision-tree algorithms. Random Forest (RF) performed best (AUC = 0.74) with final predictors of any 5 of 11 cardinal clinical depressive symptoms (e.g., anhedonia, irritable mood, sleep disturbance, suicidality) [10]. Xiang et al. compared several types of decision-tree algorithms in the ABCD sample to predict depression trajectories finding a gradient-boosted algorithm worked best (AUC = 0.90), with sleep disturbances, parent mental health burden and family financial adversity being the most important predictors [45]. In the IMAGEN cohort, Toenders et al. used LR (AUC = 0.70) and identified baseline depressive symptom severity, female sex, neuroticism, prior bullying, adverse life events, and surface area of the supramarginal gyrus as the most important predictors of later adolescent depression [14]. Finally, Huang et al. found that RF outperformed CART and SVM algorithms (AUC = 0.90), with suicidality, anhedonia, lack of social support, emotional neglect in childhood, non-suicidal self-injury and poor maternal relationship being the most important final predictors of depression in youth [11].

Anxiety has received less frequent attention than depression in terms of ML-based predictive classification. We found that deep learning produced substantially stronger performance (AUC = 0.94; Accuracy = 95%; Precision = 94%; Recall = 95%) than XGB (AUC = 0.61) or LR (AUC = 0.5), with anxiety predicted by a history of adverse life events and levels of internalizing, externalizing and emotionality traits as well as stress in the parent-child relationship and social skills. There is a similarity of our final predictors in terms of emotionality, internalizing behaviors and family-related factors with the one prior study in youth anxiety using ML-based predictive classification. Here, Chavanne et al. trained a majority voting classifier composed of RF, SVM and LR (AUC = 0.68) using 27 features (14 volumetric from structural MRI and 13 psychosocial), finding the top predictors of a pooled anxiety target (similar to our own) in future followup were neuroticism, hopelessness, emotional symptoms, family, alcohol consumption and distress level [15]. Overall, the best-performing models in our analyses discriminated between these internalizing conditions: there were no overlapping final predictors between anxiety and depression in this youth cohort.

Attention deficit and disruptive behaviors may similarly overlap phenomenologically and epidemiologically and are often collectively considered ‘externalizing’ disorders. As in the internalizing disorders, we identified different sets of final predictors for these conditions, though levels of externalizing and hyperactivity behaviors were shared between the best-performing models for each condition. We found there was a split in terms of best-performing algorithm, with attention deficit best predicted by deep learning (AUC = 0.95; Accuracy = 96%; Precision = 89%; Recall = 90%) using hyperactivity, parent feelings about their child, handedness, level of stress in the parent-child relationship, autism and externalizing traits while XGB performed best in predicting disruptive behaviors (AUC = 0.96; Accuracy = 97%; Precision = 28%; Recall = 91%) with levels of behavioral impairment, externalizing and hyperactivity traits. Similar to depression, XGB exhibited relatively low precision in predicting disruptive behaviors, so the final predictors identified by deep learning (AUC = 0.73; Accuracy = 85%; Precision = 85%; Recall = 94%) of externalizing and hyperactivity trait level, social responsiveness and mood symptom level are worthy of consideration.

Our present study identified final predictors of attention deficit and disruptive behaviors that are congruent with prior ML-based work in highlighting communication deficits and social/emotional development (likely represented in our experiments by autism trait level) and factors related to the familial/home environment.

Historical ML-based predictive modeling in ADHD has typically involved smaller sample sizes and many studies have lacked a held-out test set [16]. More recently, however, several studies have appeared on ADHD in large, cross-sectional samples, including the ENIGMA cohort (created to address the former issue) with neuroimaging. In a Swedish registry (*n* > 200,000), Garcia-Argibay et al. compared deep learning with RF, XGB Naïve Bayes and LR with the ElasticNet using 22 psychosocial features to predict ADHD cases. Deep learning performed best (AUC = 0.75), with having a relative with criminal conviction(s), sex, school performance, speech disability and acute stress level being the most important predictors [12]. Maniruzzaman et al. used the Japan National Survey (*n* > 45,000) and compared several decision-tree algorithms with K nearest neighbors, SVM and two deep learning algorithms (Multi Layer Perceptron (MLP); Convolutional Neural Net) using 19 psychosocial predictors, identifying RF as the top performer (AUC = 0.94) with child’s age, sex, mother’s age, allergies, asthma, family structure and psychiatric comorbidities as final predictors [46]. In the ENIGMA dataset, Zhang-James et al. compared the predictive performance of nine algorithms, including multiple types of decision-tree and linear algorithms and two variants of an MLP using only neural features derived from a dimensionality-reducing principal factors factor analysis. Deep learning performed best (AUC = 0.64), though the features post dimensionality reduction were not conventionally interpretable [16]. Finally, Ter-Minassian et al. used the UK Maudsley & National Pupil Database (*n* > 55,000) to compare deep learning (MLP) with RF, NB, LR and SVM and 68 psychosocial features, identifying school attendance, social/emotional development, writing performance, male sex and problem solving/reasoning [13]. This was one of the few studies in child mental health that found LR outperformed (AUC = 0.72). Several ML predictive studies have been conducted on disruptive behaviors. Menon and Krishnamurthy used diffusion, structural and resting-state imaging in 1100 youth from the ABCD cohort and compared individual vs ensemble CNN to achieve an AUC of 0.74 in predicting DBD [47]. In a particularly interesting study, Chan et al. used the first wave of the ABCD study (children aged 8–10) to predict conduct disorder using 52 features (psychosocial, prior ADHD/ODD diagnosis, resting-state graph neural metrics) with a neural network algorithm, achieving AUC = 0.91. Here, a model combining psychosocial with imaging metrics outperformed either alone, with the most important final predictors being lower parental monitoring, more aggression in the household, lower income, greater ADHD and ODD symptoms, worse crystallized cognition and card sort performance and topologic disruptions in subcortical and frontoparietal networks [8].

Our analysis of traumatic symptoms was more exploratory. The vast majority of ML-based studies aiming to predict PTS and PTSD are conducted in adult populations who have experienced military-, disaster- or medical-related traumatic events. In a 2020 comprehensive survey of the literature, Ramos-Lima et al. identified 49 such studies. Of these, only a single study was conducted with youth participants, with one additional study in youth more recently being published [48–50]. Both extant studies in youth focus on cohorts constructed to specifically address PTS after medical injury and/or hospitalization. Thus, our study represents the first to attempt prediction in a naturalistic cohort where PTS is much more likely attributable to the cumulative burden of adverse life events, including emotional trauma. In our exploratory analysis, we found PTS slightly more challenging to predict. While the best-performing algorithm proved to be XGB (AUC = 0.97; Accuracy = 100%; Precision = 5%; Recall = 94%), this technique was weak in precision, which we also found to be the case in other conditions and indeed, is a common feature of decision-tree techniques in tabular data and can be seen in peer studies reviewed above. Unfortunately, given the weakness of the deep learning and logistic regression models (AUC = 0.5), we could not turn to the former to access results with higher precision as were available for the other four conditions. XGB used final predictors of daily life function level, degree of normal development and levels of coping skills and mood symptoms. While no prior ML-based studies are available for direct comparison, these final predictors are concordant with prior work using correlative techniques to make group comparisons in youth PTS, which have found that lower intelligence/developmental level, thought suppression (a maladaptive coping skill) and poor life/family functioning have medium to large effect sizes [51]. PTS and PTSD are complex phenomena and a very active area of research. Our results are promising, but model weaknesses may reflect a lack of PTS-specific candidate predictors in the underlying data (e.g., intentionality of trauma; group versus individual trauma) or sample construction issues. In particular, we note that the randomization process used to assign cases to train and test partitions in the current analysis produced an undesirably small number of PTS cases in the test partition.

To our knowledge, this is the first study to compare the relative predictive ability of ML algorithms from different classes across multiple major mental health conditions in youth within the same analytic design, allowing us to make a number of further observations. We found that across all five conditions, psychosocial and psychometric constructs were more important final predictors than neural metrics of brain function. Our experience is largely congruent with prior research in mental health in youth and recent large-scale research demonstrating that the effect size of associations between inter-individual differences in brain structure or function and complex cognitive or mental health phenotypes are smaller than previously thought and prone to replication failures [52]. Only a small number of predictive studies in youth mental health conditions have similarly incorporated multidomain candidate predictors including neural metrics and results have been mixed. In depression, Xiang et al. found that psychosocial predictors outperformed a wide variety of neural metric types in predicting trajectory severity in the ABCD cohort, whereas Toenders et al. found that two structural MRI metrics were useful in predicting depression in the IMAGEN cohort but not as important as five other psychosocial predictors. In anxiety, Chavanne et al. found that structural MRI volumetrics did not improve the prediction of a pooled anxiety target in the IMAGEN cohort, with every psychosocial predictor except extraversion outranking volumetrics, though neural metrics made a partial contribution to predicting Generalized Anxiety Disorder. Chan et al. used the first wave of the ABCD study (children 8–10) to predict conduct disorder and found that combining psychosocial with imaging metrics outperformed either alone. However, such results in predictive classification (which, when successful, typically identifies final predictors of large importance) do not necessarily downgrade the role of neural metrics in probing disease mechanisms, particularly when specialized models allowing for the interaction of many effects of smaller size are constructed. Moreover, there is a paucity of work in predictive classification in youth incorporating multidomain data, and most extant work, including the present study, employs metrics from a single type of neuroimaging (volumetric or functional). Future studies incorporating multiple types of neural metrics with psychosocial and cognitive candidate predictors are indicated to improve our understanding of the former’s potential role in predictive classification in youth mental health.

In terms of algorithm types, we found that deep learning with ANNs or tree-based learning with XGB outperformed logistic regression across all five mental health conditions. This result is concordant with the vast majority of prior comparable studies in youth that have compared across these algorithm categories in a single disorder (see above). Further, we found that demographic characteristics with historical associations with relative severity and incidence, such as race/ethnicity and, in particular, sex/gender, where these associations might reflect bias in case ascertainment, did not prove to be shared or specific predictors of major mental illnesses in youth in the best-performing models. With scattered exceptions, this finding is also concordant with prior studies predicting these conditions in youth [12, 13, 46]. As well, it provides some empirical support that bias in case ascertainment attributable to characteristics such as race/ethnicity or sex/gender was not present. However, we also caution that this was a community-based, naturalistic sample from the NYC area only and therefore larger conclusions may not be drawn.

AI techniques are well-suited to optimizing ML predictive models in multidomain biomedical ‘big data’ where underlying mechanisms are poorly defined and likely multifactorial and/or non-linear or non-hierarchical. Notwithstanding its reputation for automation and large scale, the majority of academic ML research proceeds idiosyncratically at a small scale with ≤50 model fits explored [23]. This cramps the scientific search space, increases the potential for bias and noise and reduces statistical power. Here, we applied IEL to address prominent and current issues in the ML field of interpretability, feature selection and hyperparameter tuning and fit ~40,000 models for each condition over the course of training to generate rigorous predictions that generalized well to unseen, held-out data with algorithms that are readily available as open-source code. Our results highlight the value of investment in a principled approach to model tuning, such as IEL, in the large multidomain open science datasets that are increasingly the focus of psychiatry. Future directions may extend the approaches and findings in the present paper to large, longitudinal participant samples underway, such as the ABCD study, to mitigate the present reliance on cross-sectional assessment. Our own such study is currently in progress.

## Limitations

This study applies AI/ML predictive analytics to a cross-sectional sample of youth aged 5–21 and throughout the term ‘predict’ is used in the ML sense to mean the prediction of a class label for given examples of input data or the construction of a discriminative classifier. Neither causality nor the prediction of class labels in the future for the same example subjects, the prediction of future occurrence of cases for subjects not yet exhibiting symptoms should or can be imputed from this analysis. The participant sample in the present study was a naturalistic, community-based, undifferentiated population of youth and their parents. The present authors did not collect the original data and are unable to control for any bias that was present in data collection. No attempt was made to stratify the sample along demographic or other characteristics, and all participants were from the NYC area. Accordingly, results may not be applicable to other types of population samples from different regions or stratified samples. Further, no attempt was made to collect the original data to determine the point of disease onset, and this is a cross-sectional sample. Therefore candidate predictors have an undefined chronologic relationship to disease onset. We acknowledge that the present study does not include exhaustive connectomic or genomic data, albeit high-fidelity predictions were obtained without them. Future work might explore the possibility that including other data types could incrementally improve accuracy, sensitivity and specificity at the margin and should validate findings in an external dataset.

## Conclusion

By applying AI-optimized ML to a transdiagnostic, multidomain dataset within a common analytic architecture, we were able to delineate discrete sets of final predictors for the five most common mental illness conditions in youth with models that generalized well (AUC ≥ 0.94) in unseen, held-out test data. Our results support the current intuition that peri-adolescent psychiatric conditions are multifactorial disease processes with non-linear relationships among predictors, given the preference for ANN and XGB algorithms. In the present dataset, we found that psychosocial and psychometric predictors were preferred over metrics of neural function.

### Supplementary information

Supplementary Table 1

Supplementary Table 2

Supplementary Table 3

Supplementary Table 4

Supplementary Table 5

Supplementary Table 6
