# Supplementary material for: Predicting individual cases of major adolescent psychiatric conditions with artificial intelligence
Source: Transl Psychiatry. 2023 Oct 10;13:314. doi: 10.1038/s41398-023-02599-9 (PMC10564881; doi:10.1038/s41398-023-02599-9)
Supplement: Supplementary file 1 — Supplementary Table 1 [file 41398_2023_2599_MOESM1_ESM.docx]

**Supplementary Table 1**

Phenotypic and neural feature predictors with brief description of their contents. For the neural metric of intrinsic network connectivity, the individual metrics represent the computed pearson correlation between the two intrinsic functional networks of interest. There are two separate visual intrinsic networks.

| **Measure** | **Description** | **Detail** |
| --- | --- | --- |
| ESPAD | Drug and alcohol screening | Ever used marijuana |
| ESPAD | Drug and alcohol screening | Computed composite score of all substances |
| PhenX Neighborhood | Feelings about neighborhood safety |  |
| Age | Age at assessment |  |
| Sex | Sex at birth |  |
| PPS | Interview based puberty rating scale |  |
| FSQ | Financial support questionnaire | Type of health insurance |
| FSQ | Financial support questionnaire | Parent currently employed |
| FSQ | Financial support questionnaire | Annual household income |
| MRI site | Site at which MRI performed |  |
| PBQ | Pregnancy and birth questionnaire | Mother ever smoked |
|  | Pregnancy and birth questionnaire | Mother ever smoked during pregnancy |
|  | Pregnancy and birth questionnaire | Mother had medical problem requiring treatment |
|  | Pregnancy and birth questionnaire | Mother took prescribed medication during pregnancy |
|  | Pregnancy and birth questionnaire | Mother took illicit drugs during pregnancy |
|  | Pregnancy and birth questionnaire | Mother consumed alcohol during pregnancy |
|  | Pregnancy and birth questionnaire | Child birth weight |
| Pre-Interview Demographics | Race | African American |
| Pre-Interview Demographics | Race | Caucasian |
| Pre-Interview Demographics | Race | Hispanic |
| Pre-Interview Demographics | Race | Other |
| Pre-Interview Developmental History |  | Birth complications |
| Pre-Interview Developmental History |  | Infant temperament |
| Pre-Interview Developmental History |  | Normal development |
| Pre-Interview Developmental History |  | Growth concerns |
| Pre-Interview Developmental History |  | Newborn problems |
| Pre-Interview Developmental History |  | History of prematurity |
| Pre-Interview Developmental History |  | Mother’s age at birth |
| Pre-Interview Developmental History |  | Skill loss prior to age 6 |
| Pre-Interview Developmental History: | Pregnancy symptoms | Maternal emotional issues |
| Pre-Interview Developmental History: | Pregnancy symptoms | Threatened miscarriage |
| Pre-Interview Developmental History: | Pregnancy symptoms | Family stress |
| Pre-Interview Developmental History: | Pregnancy symptoms | Flu or other virus |
| Pre-Interview Educational History |  | Early Intervention |
| Pre-Interview Educational History |  | Individualized Education Plan |
| Pre-Interview Educational History |  | Neuropsychological Testing |
| Pre-Interview Educational History |  | After school teams |
| Pre-Interview Educational History |  | Has a best friend |
| Pre-Interview Educational History |  | Family is religious |
| Pre-Interview Educational History |  | Learning disability |
| Pre-Interview Educational History |  | Gets along with kids at school |
| Pre-Interview Educational History |  | Bullied |
| Pre-Interview Educational History |  | Get along with kids in neighborhood |
| Pre-Interview Educational History |  | Number of friends |
| Pre-Interview Educational History |  | Discipline problems |
| Pre-Interview Treatment History |  | History of emergency room visits |
| Pre-Interview Treatment History |  | Firearms in home |
| Pre-Interview Treatment History |  | History of head injuries |
| Pre-Interview Treatment History |  | History of health (medical) problems |
| Pre-Interview Treatment History |  | Currently takes psychiatric medications |
| Pre-Interview Treatment History |  | Has taken psychiatric medications in the past |
| Pre-Interview Treatment History |  | History of (physical) trauma |
| Pre-Interview Treatment History |  | Homicidal thoughts or actions |
| Pre-Interview Treatment History |  | Has been hospitalized |
| CSSRS | Severity of suicidal thoughts and behaviors | Ever wished dead or to not wake up |
| IAT | Behaviors associated with internet use | Total score |
| PCIAT | Compulsive use of internet | Total score |
| Connors | ADHD rating scale | Subscale scores |
| Connors | ADHD rating scale | Parent negative impression of child |
| Connors | ADHD rating scale | Parent positive impression of child |
| CCSC | Child’s coping strategies | Computed composite score |
| CPIC | Child’s perception of parental conflict |  |
| PSI | Stress in parent-child system |  |
| ICU | Callous and unemotional traits |  |
| SCQ | Nonverbal social communication |  |
| SDQ | Behavioral screening questionnaire | Hyperactivity traits |
| SDQ | Behavioral screening questionnaire | Internalizing traits |
| SDQ | Behavioral screening questionnaire | Prosocial traits |
| SDQ | Behavioral screening questionnaire | Externalizing traits |
| CTOPP | Phonological processing |  |
| Pegboard | Test of manipulative dexterity | Dominant hand |
| Pegboard | Test of manipulative dexterity | Nondominant hand |
| Physical assessment |  | Diastolic blood pressure |
| Physical assessment |  | Body Mass Index |
| SRS | Autism-oriented measure of interpersonal, communication skills and repetitive behaviors | Total score |
| SWAN | Parent report of psychopathology | Hyperactivity scale |
| SWAN | Parent report of psychopathology | Internalizing trait scale |
| TOWRE | Pronunciation of printed words |  |
| Full Scale Intelligence Quotient | Composite measure of general intelligence |  |
| WIAT | Achievement skills assessment | Listening comprehension |
| WIAT | Achievement skills assessment | Numerical operations |
| WIAT | Achievement skills assessment | Reading comprehension |
| FGC | Physical fitness test |  |
| SAS | Social aptitude |  |
| NLES | Family, community and school stressors and their effects |  |
| BIA | Bioelectric impedance analysis | Daily energy expenditure |
| CIS | Function in relationships, psychopathology, school and leisure |  |
| MFQ | Frequency of mood and feeling states |  |
| NIH Toolbox | Cognitive function test | Card sort score |
| NIH Toolbox | Cognitive function test | Flanker score |
| NIH Toolbox | Cognitive function test | List pattern score |
| NIH Toolbox | Cognitive function test | Pattern recognition score |
| Temporal Discounting | Delay discounting task | Run 1 |
| Temporal Discounting | Delay discounting task | Run 2 |
| PANAS | Mood scale | Negative Affect score |
| PANAS | Mood scale | Positive Affect score |
| ASSQ | Symptom checklist for autism |  |
| Barratt | Measure of social status | Total educational score |
| Barratt | Measure of social status | Total occupational score |
| DTS | Distress tolerance |  |
| RBS | Breadth of repetitive behaviors |  |
| Edinburgh Handedness Scale | Dominance of right or left hand |  |
| GFTA | Spontaneous and imitative articulation of consonant sounds | Sounds in words score |
| APQ | Parenting style and behaviors |  |
| CGAS | Global assessment of function |  |
| Neural metric | Intrinsic network connectivity | R Frontopariental x Sensorimotor |
| Neural metric | Intrinsic network connectivity | Right Auditory/Language x Sensorimotor |
| Neural metric | Intrinsic network connectivity | Left Frontoparietal x Sensorimotor |
| Neural metric | Intrinsic network connectivity | Cerebellar x Sensorimotor |
| Neural metric | Intrinsic network connectivity | Posterior Default Mode x Sensorimotor |
| Neural metric | Intrinsic network connectivity | Visual x Sensorimotor |
| Neural metric | Intrinsic network connectivity | Visual x Sensorimotor |
| Neural metric | Intrinsic network connectivity | Anterior Default Mode Network x Sensorimotor |
| Neural metric | Intrinsic network connectivity | Left Auditory/Language x Sensorimotor |
| Neural metric | Intrinsic network connectivity | Right Auditory/Language x Right Frontoparietal |
| Neural metric | Intrinsic network connectivity | Left Frontoparietal x Right Frontoparietal |
| Neural metric | Intrinsic network connectivity | Cerebellar x Right Frontoparietal |
| Neural metric | Intrinsic network connectivity | Posterior Default Mode x Right Frontoparietal |
| Neural metric | Intrinsic network connectivity | Visual x Right Frontoparietal |
| Neural metric | Intrinsic network connectivity | Visual x Right Frontoparietal |
| Neural metric | Intrinsic network connectivity | Anterior Default Mode Network x Right Frontoparietal |
| Neural metric | Intrinsic network connectivity | Left Auditory/Language x Right Frontoparietal |
| Neural metric | Intrinsic network connectivity | Left Frontoparietal x Right Auditory/Language |
| Neural metric | Intrinsic network connectivity | Cerebellar x Right Auditory/Language |
| Neural metric | Intrinsic network connectivity | Posterior Default Mode x Right Auditory/Language |
| Neural metric | Intrinsic network connectivity | Visual x Right Auditory/Language |
| Neural metric | Intrinsic network connectivity | Visual x Right Auditory/Language |
| Neural metric | Intrinsic network connectivity | Anterior Default Mode Network x Right Auditory/Language |
| Neural metric | Intrinsic network connectivity | Left Auditory/Language x Right Auditory/Language |
| Neural metric | Intrinsic network connectivity | Cerebellar x Left Frontoparietal |
| Neural metric | Intrinsic network connectivity | Posterior Default Mode x Left Frontoparietal |
| Neural metric | Intrinsic network connectivity | Visual x Left Frontoparietal |
| Neural metric | Intrinsic network connectivity | Visual x Left Frontoparietal |
| Neural metric | Intrinsic network connectivity | Anterior Default Mode Network x Left Frontoparietal |
| Neural metric | Intrinsic network connectivity | Left Auditory/Language x Left Frontoparietal |
| Neural metric | Intrinsic network connectivity | Posterior Default Mode x Cerebellar |
| Neural metric | Intrinsic network connectivity | Visual x Cerebellar |
| Neural metric | Intrinsic network connectivity | Visual x Cerebellar |
| Neural metric | Intrinsic network connectivity | Anterior Default Mode Network x Cerebellar |
| Neural metric | Intrinsic network connectivity | Left Auditory/Language x Cerebellar |
| Neural metric | Intrinsic network connectivity | Visual x Posterior Default Mode |
| Neural metric | Intrinsic network connectivity | Visual x Posterior Default Mode |
| Neural metric | Intrinsic network connectivity | Anterior Default Mode Network x Posterior Default Mode |
| Neural metric | Intrinsic network connectivity | Left Auditory/Language x Posterior Default Mode |
| Neural metric | Intrinsic network connectivity | Visual x Visual |
| Neural metric | Intrinsic network connectivity | Anterior Default Mode Network x Visual |
| Neural metric | Intrinsic network connectivity | Left Auditory/Language x Visual |
| Neural metric | Intrinsic network connectivity | Anterior Default Mode Network x Visual |
| Neural metric | Intrinsic network connectivity | Left Auditory/Language x Visual |
| Neural metric | Intrinsic network connectivity | Anterior Default Mode Network x Left Auditory/Language |
| Neural metric | Dynamism | Number of brain states traversed |
| Neural metric | Dynamism | Number of times subject switches between brain states |
| Neural metric | Dynamism | Maximal L^1^ span achieved between brain states |
| Neural metric | Dynamism | Total distance ‘traveled’ through brain state space (Sum of all L^1^ distances) |
| Neural metric | DVARS statistic | Head motion |
