# Supplementary material for: Predicting individual cases of major adolescent psychiatric conditions with artificial intelligence
Source: Transl Psychiatry. 2023 Oct 10;13:314. doi: 10.1038/s41398-023-02599-9 (PMC10564881; doi:10.1038/s41398-023-02599-9)
Supplement: Supplementary file 5 — Supplementary Table 5 [file 41398_2023_2599_MOESM5_ESM.docx]

**Supplementary Table 5: Hyperparameters tuned via Integrated Evolutionary Learning**

| **Algorithm type and hyperparameters** | **Range** | **Mutation Shift** |
| --- | --- | --- |
| **Artificial neural network**  Learning rate  Beta 1  Beta 2 | 0.00001-0.01 0.9-0.999 0.9-0.999 | 0.0001 0.001 0.001 |
| **XGBoost (tree-based)**  Maximum tree depth  Node partition threshold (gamma)  L1 penalty (alpha) | 2-10 0-0.00001 0.1-0.9 | 1 0.0000001 0.001 |
| **ElasticNet (linear)**  L1 penalty  L2 penalty | 0-1 0-1 | 0.01 0.01 |
